# Metals Ions Removal by Polymer Membranes of Different Porosity

**DOI:** 10.1155/2013/957202

**Published:** 2013-05-28

**Authors:** Katarzyna Jasiewicz, Robert Pietrzak

**Affiliations:** Laboratory of Applied Chemistry, Faculty of Chemistry, Adam Mickiewicz University in Poznań, Umultowska 89b, 61-614 Poznań, Poland

## Abstract

The effect of the amount of pore generating agent (polyvinylpyrrolidone) added to standard polymer membranes containing 18 wt.% of polyethersulfone on the physicochemical properties of the membranes and their capacity for removal of iron and copper ions from the liquid phase was studied. The membranes were obtained by the phase inversion method. The results have shown that the modification of polymer membranes by the use of different amounts of the pore forming agent in their syntheses leads to significant changes in porosity and has beneficial effect on equilibrium water content. The membranes studied were found to show different acid-base surface character, but for all membranes studied, a significant dominance of oxygen groups of acidic character was evidenced. The most effective were the membranes of the lowest content of polyvinylpyrrolidone, while the lowest values of resistance showed the membranes of the highest content of PVP, and so the ones of the greatest porosity.

## 1. Introduction

Pollution of the natural environment with heavy metals is to a great degree related to man's activity and has become a serious problem all over the world. The main sources of polluted water are households, metallurgic industry, and coal power plants [1]. Heavy metal ions are easily assimilated by a human organism and even when present in low concentrations can cause serious health problems. Because of their adverse effect, the rules regarding the environment protection have become even more restrictive [2].

Removal of heavy metals such as copper, iron, cadmium or zinc is done from water solutions by different methods of separation including precipitation, coagulation, ion exchange, adsorption, and membrane technology [3]. The latter have become increasingly popular because of their many advantages such as high effectiveness of pollutants removal, low energy consumption, or a possibility of use in the high flow rate conditions [4, 5].

The most important for the process are the membranes, which have recently been produced from polymer material. Theoretically each polymer can be used as membrane material, but in practice, only a restricted group of polymers have been used of certain chemical and physical properties [6]. Because of its very attractive properties, very often polyethersulfone is used [7]. It shows good thermal stability, high mechanical resistance, chemical neutrality, wide range of tolerated pH, and physiological neutrality [8]. The problem is its high hydrophobicity, which leads to deposition of particles on the surface or in membrane pores leading to its fouling and consequently to reduced flow and membrane lifetime. In order to increase the surface hydrophilicity, different modifications can be applied at the stage of membrane synthesis process [9]. The modifying agent added to the polymer solution [10] included surfactant [11], mineral filler [12], or nonsolvent [13]. These substances were added to increase hydrophilicity, increase or decrease the number of generated macropores, and increase the number of pores and improve connections between them [14].

This study was undertaken to establish the effect of a pore generating agent (PVP) added in different amounts to polymer membranes based on polyethersulfone on the membrane properties. The performance of PVP modified membranes in removal of copper and iron ions from liquid phase was analysed.

## 2. Experimental

### 2.1. Materials

Polyethersulfone (Ultrason E 6020 PES) was purchased from BASF and used as a membrane material. 1-Methyl 2-pyrrolidone (NMP) was purchased from POCh and used without further purification. Polyvinylpyrrolidone (PVP, 10 000 g/mol) as a pore former was supplied by Sigma Aldrich.

### 2.2. Preparation of PES Membranes

Casting solutions of PES 18 wt.% and 1, 2, 3, or 4 wt.% of PVP were prepared by mixing the ingredients in a flask. The casting solution obtained was left to rest for about 12 h to allow complete release of bubbles. After that, it was cast onto a glass plate using a stainless-steel knife to get a casting film of 300 *μ*m thickness, exposed to the atmosphere for 40 s, and then immersed into a coagulation bath of pure water. The as-prepared cast solution films were immersed and kept for 24 h in a deionised water bath conditioned at 25°C to complete the exchange between the solvent and nonsolvent. Directly prior to use, each electrode was washed with a small amount of deionised water.

### 2.3. Membrane Structure Characterization

#### 2.3.1. Porosity and Equilibrium Water Content

The membrane porosity was determined by the mass loss of wet membrane after drying. The membrane sample was mopped with water on the surface and weighed under wet status. Then, the membrane sample was dried until a constant mass. The membrane porosity *ε* was evaluated from (1):

(1) $$\begin{array}{c} \varepsilon =\frac{W_{w}-W_{d}}{\rho \cdot v}\cdot 100\%, \end{array}$$

where *W*_*w*_ is the mass of a wet membrane sample, *W*_*d*_ is the mass of dry state membrane sample, *ρ* is pure water density, and *v* is the volume of a membrane in wet state.

The equilibrium water content (EWC) was determined by (2):

(2) $$\begin{array}{c} \text{EWC}=\frac{W_{w}-W_{d}}{W_{w}}\cdot 100\%. \end{array}$$

#### 2.3.2. Contact Angle

The contact angle between water and membrane was directly measured using a contact angle measuring instrument G10, KRUSS, Germany. For evaluation of the membrane hydrophilicity deionized water was used as a probe liquid in all measurements. To minimize the experimental error, the contact angle was measured at five random locations for each sample and then the average was reported.

#### 2.3.3. Surface Oxygen Groups

The surface properties were characterised using potentiometric titration experiments using 809 Titrando equipment manufactured by Metrohm. The instrument was set at the mode when the equilibrium pH was collected. Materials studied in the amount of about 0.100 g in 50 mL 0.01 M NaNO_3_ were placed in a container thermostated at 25°C and equilibrated overnight with the electrolyte solution. To eliminate the influence of atmospheric CO_2_, the suspension was continuously saturated with N_2_. The carbon suspension was stirred throughout the measurements. Volumetric standards NaOH (0.1 M) or HCl (0.1 M) were used as titrants [15].

### 2.4. Membrane Performance Characterization

Water permeability of the membranes prepared was measured in a stain less-steel cell, holding the effective membrane area of 19.6 cm^2^. The membranes were initially subjected to deionised water of 3 bars for about 1.5 h before testing. Then, the pure water flux was measured at 3 bars, 23 ± 1°C, and 0.22 m/s cross-flow velocity. The pure water flux was calculated from the following equation:

(3) $$\begin{array}{c} J_{w}=\frac{V}{A\cdot \Delta t}, \end{array}$$

where *J*_*w*_ (L/(m^2 ^h)) is the pure water flux, *V* (L) is the volume of permeated water, *A* (m^2^) is the effective membrane area, and Δ*t* (h) is the permeation time.

The experiments were conducted using compressed nitrogen gas and iron or copper solutions of different initial concentrations (5, 10, 15, or 20 mg/dm^3^), and all measurements were made at 3 bars, in triplicate. The final concentration of iron or copper in the solution was analysed using a double beam UV–Vis spectrophotometer (Varian Cary 100 Bio) at 487 nm wavelength for iron solutions and at 620 nm wavelength for copper solutions. The iron or copper rejections (%*R*) were calculated from (4):

(4) $$\begin{array}{c} \%R=\left(1-\frac{C_{p}}{C_{f}}\right)\cdot 100, \end{array}$$

where *C*_*p*_ and *C*_*f*_ (mg/mL) were iron or copper concentrations in the permeate and the feed solutions, respectively.

Membrane resistance was evaluated according to Darcy's law by the resistance in the series of models as follows:

(5) $$\begin{array}{c} J=\frac{\Delta P}{\mu R_{t}}, \end{array}$$

where *J* (L/(m^2 ^h)) is the permeate flux, Δ*P* is the transmembrane pressure (TMP), *μ* is the dynamic viscosity of permeate, and *R*_*t*_ is the total filtration resistance. The resistance in the series of models combines various levels resistance causing flux decline as follows:

(6) $$\begin{array}{c} R_{t}=R_{m}+R_{p}+R_{c}, \end{array}$$

where *R*_*t*_ is the total filtration resistance composed of various levels resistance including that of the membrane itself *R*_*m*_, pore blocking *R*_*p*_, cake resistance *R*_*c*_. The intrinsic membrane resistance (*R*_*m*_) can be estimated from the initial pure water flux (3). Fouling resistance (*R*_*p*_) is caused by pore plugging and irreversible adsorption of foulants on membrane pore wall or surface. Cake resistance (*R*_*c*_) induced by cake layer formed on the membrane surface was calculated from the water flux after pure water washing [16, 17].

The detailed membrane fouling behaviour was studied as follows. Firstly, pure water flux of the membrane *J*_*w*1_ (L/(m^2 ^h)) was tested at 3 bars. Then, aqueous solution of iron or copper (5–20 mg/dm^3^) was fed into the ultrafiltration system. After filtration for 30 min, the membrane was flushed with pure water for 10 min and then pure water flux of the membrane *J*_*w*2_ (L/(m^2 ^h)) was measured. The flux recovery ratio (FRR) was calculated using (5) to evaluate membrane antifouling property:

(7) $$\begin{array}{c} \text{FRR}\left(\%\right)=\frac{J_{w2}}{J_{w1}}\cdot 100\%. \end{array}$$

## 3. Results and Discussion

### 3.1. Membrane Characterisation

Table 1 presents the data on porosity, equilibrium water content, and contact angles obtained for the studied series of PES-18 membranes.

The results show that the number of pores in the membranes containing 2 and 3 wt.% of PVP is smaller than that in samples PES-18 PVP-1 and PES-18 PVP-4. The membrane of the greatest addition of PVP is characterised by the highest number of pores. With increasing amount of PVP added, the equilibrium content of water increased. A similar relation was observed for the contact angle. The membrane wettability was measured on both active and inactive surfaces. The contact angle values measured on the inactive surface of the membrane were smaller than those on the active surface. Membrane PES-18 PVP-1 showed the greatest hydrophilicity, while the membrane with the greatest content of PVP revealed the lowest hydrophilicity from all samples in the series studied.

### 3.2. Membrane Performance

**Figure 1:**
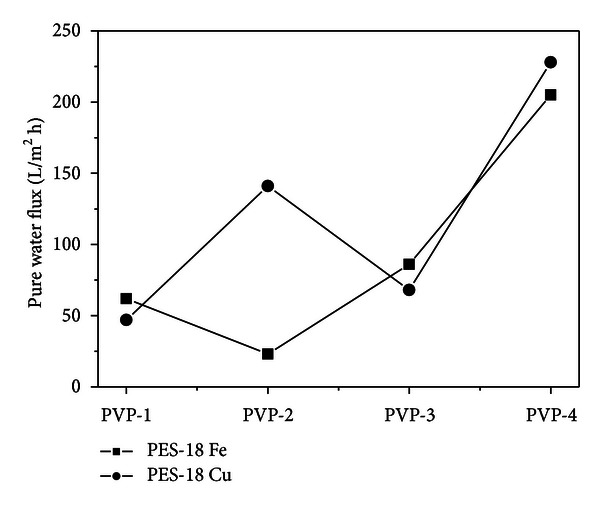
Pure water flux of the membranes studied.

Figure 1 presents the pure water flux measured for the series of PES-18 membranes of different porosity after the filtration of iron and copper ions solutions. As follows from these data, with increasing content of pore generating agent, the pure water flux increases for the membranes after filtration of iron ions. The exception is membrane PES-18 PVP-2 for which the pure water flux measured was much lower than those for the other membranes. Such a direct relationship between the pore generating agent content and pure water flux could not be found for the membranes used for filtration of copper solution. However, for both series of membranes, the highest pure water flux was measured for the samples with the greatest content of pore generating agent. 

To evaluate the character of the membrane surfaces, the number of oxygen functional groups was measured for the membranes studied before and after the filtration of copper solution. According to the results presented in Tables 2 and 3, for the membranes containing 2 and 3 wt.% of PVP, the number of oxygen groups of acidic character increased after filtration of copper solution, especially those of pKa = 3–5 and pKa > 11. However, the number of oxygen groups of acidic character of pKa from the same ranges decreased after filtration for membranes PES-18 PVP-1 and PES-18 PVP-4. After filtration of copper solution through the membranes containing 1 or 3 wt.% of PVP, the acidic groups of pKa = 5–7 disappear. However, for PES-18 PVP-1, the acidic groups of pKa = 9–11 appear. For the membranes containing 2 or 4 wt.% of PVP, also a small increase in the content of oxygen functional groups of acidic character (pKa = 9–11) is noted. As far as the content of oxygen basic groups is concerned, (Table 3), their number increases after filtration for all membranes except PES-18 PVP-4. For the membranes containing 1, 2, or 4 wt.% of PVP, a considerable increase in the number of oxygen surface basic groups of pKa = 3–5 was observed after filtration of copper solution. For PES-18 PVP-3, the content of surface basic groups of pKa = 3–5 remains at a similar level after filtration. The content of oxygen groups of basic character of pKa = 9–11 increases for the membranes containing 1, 2, and 3 wt.% of PVP, while it decreases for the membrane with 4 wt.% of PVP.

The contents of surface oxygen groups on the membrane surfaces before and after filtration of iron solution (15 mg/dm^3^) are given in Tables 4 and 5. According to the total content of surface oxides and contents of acidic and basic groups, on the surfaces of all the membranes studied, before and after filtration of iron solution, the groups of acidic character dominate.

As far as acidic groups are concerned, (Table 4), on the surfaces of all membranes studied, both before and after iron solution filtration, the dominant groups are those of pKa = 3–5 and >11. It should be noted that after the filtration, the content of acidic groups of the previously mentioned pKa values increases, except for membranes PES-18 PVP-1. 

As to the oxygen groups of basic character, (Table 5), prior to filtration, their amounts are similar for all membranes studied. The only exception is membrane PES-18 PVP-2, in which the content of surface basic oxygen groups is close to 0.9 mmol/g. Moreover, for all membranes, the clearly dominant basic oxygen groups are those of pKa = 9–11. 

Detailed analysis of individual oxygen groups (Table 4) has revealed that after the filtration of iron solution, the number of acidic groups of pKa = 3–5 increases, which is particularly pronounced for the membranes containing 2 and 3 wt.% PVP. Only for membrane PES-18 PVP-1, the content of acidic groups of PKa = 3–5 considerably decreases. Moreover, for the membranes containing 1 and 3 wt.% PVP, the surface oxygen groups of pKa = 5–7 disappear, while those of pKa = 9–11 appear. For membranes PES-18 PVP-1, 3, and 4, after the filtration of iron solution, the number of oxygen acidic groups of pKa > 11 decreases, while that of the surface groups of pKa = 9–11 increases. For membrane PES-18 PVP-2, the number of surface oxygen groups of pKa from both above ranges increases, while for PES-18 PVP-4 the number of oxygen surface groups of pKa = 9–11 increases and for that of pKa > 11 it decreases. After filtration of an iron solution, the number of basic surface oxygen groups of pKa = 9–11 significantly increases (Table 5). Moreover, for PES-18 PVP-2 after filtration, a small increase is noted in the number of basic surface oxygen groups of pKa = 3–7 and pKa > 11. For the other membranes, the content of these groups is at a similar level.

**Figure 2:**
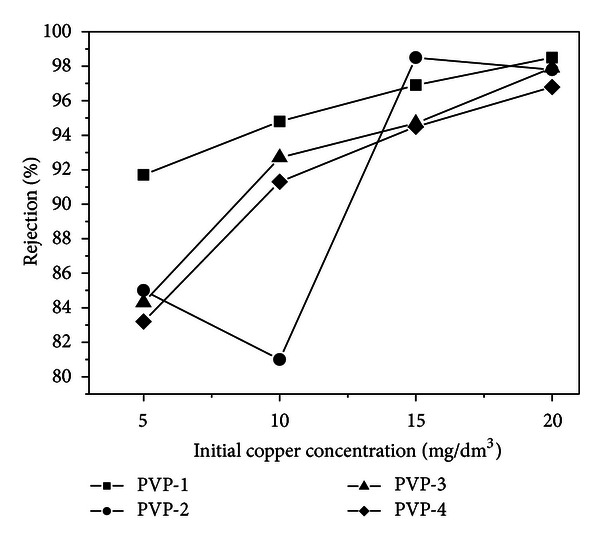
Copper rejection (*R*) versus the initial copper concentration in the solution used for filtration with PES-18 membranes.

The efficiency of copper ions removal by the membranes studied in the form of the iron or copper rejections versus the initial concentration of the feed solution is presented in Figure 2. For the membranes containing 1, 3, and 4** **wt.% PVP, the efficiency curves have similar character and the membrane efficiency increases with increasing concentration of the copper or iron feed solution. However, with increasing content of pore generating agent (PVP), the efficiency of copper ions removal decreases. The membrane of the lowest pore-generating agent revealed the highest efficiency of metal ions removal, for metal ions solutions of all concentrations studied. For the membrane containing 2** **wt.% PVP, the relation between efficiency and concentration of copper or iron feed solution is unique. For this membrane, the efficiency of copper ion removal for the feed solution of the lowest (5** **mg/dm^3^) and the highest (20** **mg/dm^3^) concentrations is similar to that for the other membranes, but for the copper feed solutions of 10 and 15** **mg/dm^3^, the behaviour of PES-18 PVP-2 is completely different. For the feed solution of 10** **mg/dm^3^, the efficiency of this membrane is by far the lowest, while for the feed solution of 15** **mg/dm^3^, it is the highest from among all membranes studied.

**Figure 3:**
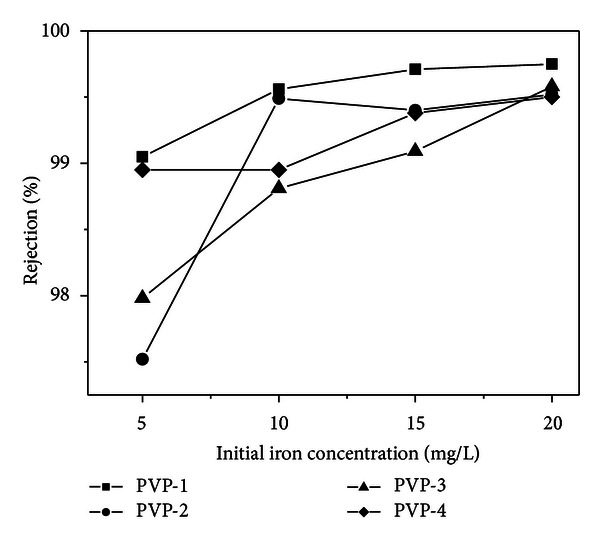
Iron rejection (*R*) versus the initial iron concentration in the solution used for filtration with PES-14 membranes.

Figure 3 presents the iron rejection for a series of membranes PES-18 versus the feed solution concentration. Similarly as for the copper feed solution, the efficiency of the membranes increases with increasing iron solution, except for membrane PES-18 PVP-2. For the whole series of membranes studied, with increasing concentration of the feed solution, the efficiency increases, and for the highest feed solution concentration, the efficiency reaches maximum values close to 99%. In general, the PES-18 series membranes are the most effective for removal of iron from the feed solutions of the highest concentrations of those considered.

**Figure 4:**
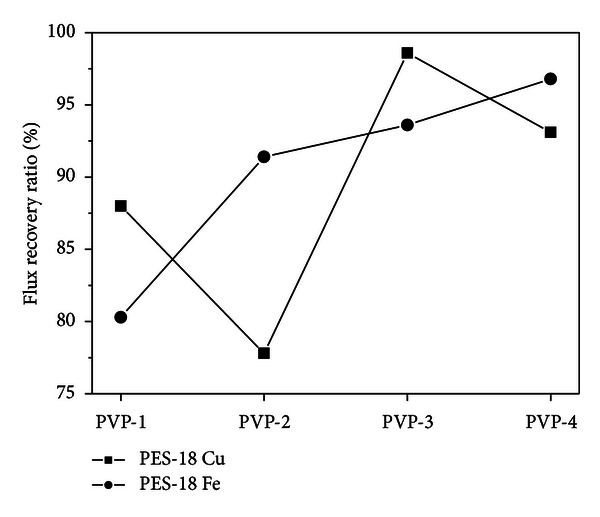
Flux recovery ratio of PES-18 series membranes after filtration of iron and copper solution.

The flux recovery ratios (FRR) obtained for the membranes after filtration of copper or ion solutions are presented in Figure 4. For the membranes of the lowest and highest contents of the pore-generating agent (PES-18 PVP-1 and PES-18 PVP-4), the FRR values after copper solution filtration are similar. The lowest FRR (77.8%) from among the whole series of membranes was obtained for PES-18 PVP-2, while the highest (98.6%)for PES-18 PVP-3, after filtration of a copper solution. After filtration of iron solution, the values of FRR increase with increasing PVP content and reach 96.8% for PES-18 PVP-4.

Table 6 presents the results concerning the resistance levels upon filtration of an iron solution of 15 mg/dm^3^. According to these data, the total filtration resistance (*R*_*t*_) decreases with increasing content of the pore generating agent, except for PES-18 PVP-2 whose total filtration resistance (*R*_*t*_) is 31,95 × 10^13^ (L/m^2 ^h) which is much higher than those obtained for the other membranes. The resistance levels of cake (*R*_*c*_) and pore blocking (*R*_*p*_) also decrease with increasing content of PVP in the membranes. For PES-18 PVP-3 and PES-18 PVP-4, the cake resistance (*R*_*c*_) is the lowest from among all membranes of the series studied.

The values of particular components of total filtrating resistance calculated for the membranes of PES-18 series upon filtration of a copper solution of 15 mg/dm^3^ are given in Table 7. For PES-18 PVP-1 and PES-18 PVP-3, the total resistance levels are higher than those for the other membranes, and the values found for PES-18 PVP-3 are the highest from among those obtained for the whole series. For the membranes containing 1, 2, and 4 wt.% PVP, the contribution of resistance particular types in the total filtration resistance can be ordered as *R*_*m*_ < *R*_*p*_ < *R*_*c*_, whereas for PES-18 PVP-3, this order is reversed. For the membrane of the highest content of PVP, the values of particular total resistance components are the lowest, similarly as for the iron solution filtration.

## 4. Conclusions

Modification of polyethersulfonic membranes by addition of polyvinylpyrrolidone (PVP) in different amounts into the membrane forming solution leads to obtaining membranes of different porosity and has a beneficial effect on the equilibrium water content. The pure water flux was the greatest for the membranes of the greatest porosity. The membranes modified with different amounts of PVP have surfaces of different acid-base character; however, the oxygen groups of acidic character dominate. The highest efficiency of filtration was found for the membranes of the lowest content of PVP, but the lowest resistance was determined for the membranes of the highest PVP content, and so the one of the highest porosity. 

## Figures and Tables

**Table 1:** Porosity (*ε*), equilibrium water content (EWC), and contact angle of investigated membranes.

Membrane	*ε* (%)	EWC (%)	Contact angle	
			Top	Bottom
PES-18 PVP-1	50.45	75.16	76.0 ± 5.10	71.2 ± 4.86
PES-18 PVP-2	45.18	76.06	76.1 ± 3.45	71.6 ± 2.16
PES-18 PVP-3	47.39	76.50	76.9 ± 3.25	71.7 ± 1.96
PES-18 PVP-4	65.90	78.67	77.2 ± 3.58	71.8 ± 1.62

**Table 2:** Acidic properties of investigated adsorbents before and after filtration with 15 mg/dm^3^ copper solution (mmol/g).

Membrane	pKa															
	<3		3–5		5–7		7–9		9–11		>11		Acidic groups		Total content of surface oxides	
	A	B	A	B	A	B	A	B	A	B	A	B	A	B	A	B
PES-18 PVP-1	—	—	3.86	2.11	1.32	—	0.14	0.08	—	1.02	1.72	0.50	7.04	3.71	8.98	6.97
PES-18 PVP-2	—	—	0.55	2.01	—	—	0.06	0.13	0.66	1.02	0.87	1.18	2.14	4.34	3.04	7.07
PES-18 PVP-3	—	—	0.58	1.85	0.98	—	0.21	0.11	—	—	1.54	2.01	3.31	3.97	5.19	5.65
PES-18 PVP-4	—	—	2.14	1.75	—	—	0.09	0.08	0.81	0.87	1.30	0.22	4.34	2.92	6.40	5.80

A: before filtration, B: after filtration.

**Table 3:** Basic properties of investigated adsorbents before and after filtration with 15 mg/dm^3^ copper solution (mmol/g).

Membrane	pKa															
	<3		3–5		5–7		7–9		9–11		>11		Basic groups		Total content of surface oxides	
	A	B	A	B	A	B	A	B	A	B	A	B	A	B	A	B
PES-18 PVP-1	—	—	0.27	1.49	0.30	0.34	—	—	0.99	1.02	0.38	0.41	1.94	3.26	8.98	6.97
PES-18 PVP-2	—	—	0.13	1.37	0.10	0.31	0.05	—	0.62	1.05	—	0.36	0.90	2.73	3.04	7.07
PES-18 PVP-3	—	—	0.26	0.25	0.24	0.24	0.18	—	0.87	1.19	0.33	—	1.88	1.68	5.19	5.65
PES-18 PVP-4	—	—	0.26	1.25	0.31	0.27	—	—	1.12	1.00	0.37	0.36	2.06	2.88	6.40	5.80

A: before filtration, B: after filtration.

**Table 4:** Acidic properties of investigated adsorbents before and after filtration with 15 mg/dm^3^ iron solution (mmol/g).

Membrane	pKa															
	<3		3–5		5–7		7–9		9–11		>11		Acidic groups		Total content of surface oxides	
	A	B	A	B	A	B	A	B	A	B	A	B	A	B	A	B
PES-18 PVP-1	—	—	3.86	2.05	1.32	—	0.14	0.15	—	0.78	1.72	1.63	7.04	4.61	8.98	6.88
PES-18 PVP-2	—	0.24	0.55	2.01	—	—	0.06	0.13	0.66	0.69	0.87	1.18	2.14	4.25	3.04	6.41
PES-18 PVP-3	—	—	0.58	1.98	0.98	—	0.21	0.08	—	0.86	1.54	0.68	3.31	3.60	5.19	5.64
PES-18 PVP-4	—	—	2.14	2.28	—	—	0.09	0.12	0.81	1.08	1.30	1.02	4.34	4.50	6.40	6.93

A: before filtration, B: after filtration.

**Table 5:** Basic properties of investigated adsorbents before and after filtration with 15 mg/dm^3^ iron solution (mmol/g).

Membrane	pKa															
	<3		3–5		5–7		7–9		9–11		>11		Basic groups		Total content of surface oxides	
	A	B	A	B	A	B	A	B	A	B	A	B	A	B	A	B
PES-18 PVP-1	—	—	0.27	0.27	0.30	0.32	—	—	0.99	1.35	0.38	0.33	1.94	2.27	8.98	6.88
PES-18 PVP-2	—	—	0.13	0.26	0.10	0.30	0.05	—	0.62	1.15	—	0.45	0.90	2.16	3.04	6.41
PES-18 PVP-3	—	—	0.26	0.23	0.24	0.26	0.18	—	0.87	1.18	0.33	0.37	1.88	2.04	5.19	5.64
PES-18 PVP-4	—	—	0.26	0.25	0.31	0.30	—	0.12	1.12	1.40	0.37	0.36	2.06	2.43	6.40	6.93

A: before filtration, B: after filtration.

**Table 6:** Filtration resistance of different membranes for 15 mg/dm^3^ iron solution.

Membrane	*R*_*m*_ (×10^13^)	*R*_*p*_ (×10^13^)	*R*_*c*_ (×10^13^)	*R*_*t*_ (×10^13^)
PES-18 PVP-1	1.41	1.70	1.94	5.05
PES-18 PVP-2	9.95	11.00	11.00	31.95
PES-18 PVP-3	1.44	1.48	1.40	4.32
PES-18 PVP-4	1.30	1.30	1.18	3.78

**Table 7:** Filtration resistance of different membranes for 15 mg/dm^3^ copper solution.

Membrane	*R*_*m*_ (×10^13^)	*R*_*p*_ (×10^13^)	*R*_*c*_ (×10^13^)	*R*_*t*_ (×10^13^)
PES-18 PVP-1	0.87	1.07	1.16	3.10
PES-18 PVP-2	0.47	0.63	0.69	1.79
PES-18 PVP-3	2.54	2.53	2.36	7.43
PES-18 PVP-4	0.31	0.36	0.42	1.09
